# CK1δ-dependent SNAPIN dysregulation drives lysosomal failure in HIV-1 Vpr-exposed neurons: A targetable mechanism in HAND

**DOI:** 10.1016/j.isci.2025.114544

**Published:** 2025-12-26

**Authors:** Bassel E. Sawaya, Maryline Santerre

**Affiliations:** 1 Molecular Studies of Neurodegenerative Diseases Lab, FELS Cancer Institute for Personalized Medicine and Department of Neurology Lewis Katz School of Medicine, Temple University, Philadelphia, PA, USA; 2 Department of Neurology Lewis Katz School of Medicine, Temple University, Philadelphia, PA, USA

**Keywords:** Biochemistry, Pharmacology, Virology

## Abstract

HIV-associated neurocognitive disorders (HANDs) persist in nearly 40% of virally suppressed individuals despite antiretroviral therapy (ART). Lysosomal dysfunction has emerged as a key contributor to HAND pathogenesis, yet the molecular mechanisms linking chronic HIV exposure to impaired neuronal degradation remain incompletely defined. Here, we identify HIV-1 viral protein R (Vpr) as a driver of lysosomal acidification failure, clustering, and degradative impairment in neurons. We report casein kinase 1 delta (CK1δ) as a central mediator of this dysfunction, acting via phosphorylation of the adapter protein SNAPIN. Vpr-induced CK1δ activation leads to hyperphosphorylation of SNAPIN, disrupting lysosomal positioning and motility. These defects are rescued by selective CK1δ inhibition, which restores lysosomal acidification, positioning, and mitophagy. Our findings define a Vpr-CK1δ-SNAPIN axis that contributes to HANDs and highlight lysosomal transport as a targetable mechanism in neurodegeneration.

## Highlights

• Vpr promotes CK1δ-dependent SNAPIN disruption and lysosomal dysfunction
• CK1δ inhibition restores lysosomal acidification, motility, and mitophagy
• SNAPIN Ser50 phosphorylation mediates Vpr-induced lysosomal clustering
• CK1δ-SNAPIN axis reveals a targetable mechanism in HAND neurodegeneration

## Graphical abstract

**Figure:**
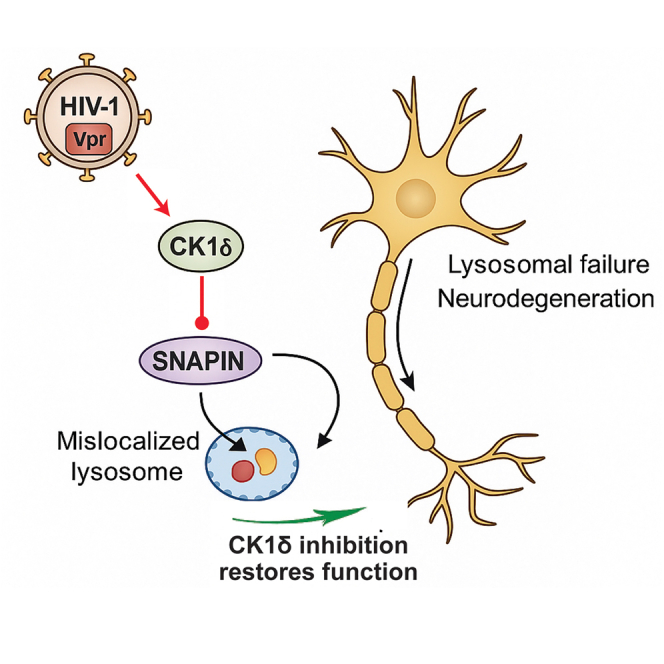

## Introduction

HIV-associated neurocognitive disorders (HANDs) remain a significant clinical challenge affecting approximately 40%–50% of people living with HIV (PLWH) despite effective viral suppression with combination antiretroviral therapy (cART).^1^ The spectrum of HAND encompasses conditions ranging from asymptomatic neurocognitive impairment (ANI) to mild neurocognitive disorder (MND) and the more severe HIV-associated dementia (HAD).^2^ While the incidence of severe dementia has declined with modern antiretroviral therapies, milder forms of cognitive impairment persist and significantly impact quality of life.^3^ The persistence of these neurological complications despite undetectable viral loads in peripheral blood suggests complex pathogenic mechanisms beyond active viral replication.^4^^,^^5^^,^^6^

Lysosomes play essential roles in neuronal homeostasis through the degradation of damaged organelles, protein aggregates, and other cellular waste products.^7^ Disruption of lysosomal acidification, positioning, or enzymatic function can lead to the accumulation of neurotoxic substrates, amplifying oxidative stress and inflammation. This phenotype is observed in Alzheimer’s disease (AD), Parkinson’s disease,^8^^,^^9^ and increasingly in HAND. Autopsy studies of HAND patients reveal characteristic morphological alterations in endolysosomes and mitochondria, suggesting that these organelles are targets of HIV-induced neuronal injury.^10^^,^^11^^,^^12^^,^^13^

HIV-1 viral protein R (Vpr) is a 96-amino acid accessory protein that continues to be detected in the cerebrospinal fluid of PLWH despite long-term antiretroviral therapy.^14^^,^^15^ Vpr exhibits remarkable functional versatility, affecting multiple cellular processes including cell cycle regulation, nuclear transport, and immune function.^16^^,^^17^^,^^18^ In the context of HIV neuropathogenesis, Vpr has been demonstrated to cause direct neurotoxicity by inducing mitochondrial dysfunction and oxidative stress.^19^^,^^20^ Furthermore, extracellular Vpr decreases neuronal ATP levels^21^ and disrupts intracellular redox balance, ultimately affecting neuronal health.^22^^,^^23^^,^^24^

Recent investigations have uncovered a previously unrecognized role of Vpr in lysosomal biology. Specifically, Vpr has been shown to impair lysosomal clearance in neurons by decreasing lysosomal acidification and deregulating lysosomal positioning.^25^ These alterations result in the accumulation of substrates like alpha-synuclein (SNCA), potentially establishing mechanistic links between HIV infection and neurodegenerative processes observed in aging and Parkinson’s disease. However, the precise molecular mechanisms by which Vpr mediates these effects on lysosomal function remain incompletely defined.

Casein kinase 1 delta (CK1δ), a serine/threonine protein kinase, regulates diverse cellular processes including membrane trafficking, circadian rhythm, cell cycle progression, and autophagy.^26^ CK1δ dysregulation has been linked to AD, where its aberrant activity alters tau phosphorylation, promotes neuroinflammation, and disrupts proteostasis.^27^

Previous studies have identified SNAPIN as an interaction partner of CK1δ through yeast two-hybrid screening.^28^ SNAPIN serves as a critical adapter protein that facilitates the recruitment of late endosomes to the dynein motor complex,^29^ enabling retrograde transport along microtubules and subsequent lysosome maturation.^30^ This process is essential for maintaining proper lysosomal positioning and function in neurons, where polarized trafficking must occur over considerable distances.^31^^,^^32^^,^^33^ Dysregulation of this trafficking axis could therefore compromise neuronal clearance mechanisms in both infectious and non-infectious neurodegenerative contexts.

Phosphorylation events tightly regulate SNAPIN’s interaction with the dynein motor complex^34^ and consequently affect lysosomal trafficking.^35^ Multiple kinases, including DYRK3^36^ and p38α-MAPK,^37^ have been shown to phosphorylate SNAPIN and modulate its functions. SNAPIN also interacts with components of the BORC/BLOC-1 complexes, multisubunit assemblies that regulate lysosomal positioning and the biogenesis of lysosome-related organelles.^38^^,^^39^ Disruption of these interactions can lead to aberrant lysosomal distribution and compromised degradative capacity.

Mitophagy, the selective degradation of damaged mitochondria by autophagy, represents an essential quality control mechanism in neurons.^40^ HIV infection has been shown to alter mitochondrial dynamics in the CNS, with evidence suggesting that imbalances in mitochondrial fission/fusion contribute to neurodegeneration in HANDs.^41^

Given the established roles of both Vpr and the CK1δ-SNAPIN axis in lysosomal positioning and function, along with the known impact of HIV infection on mitochondrial quality control processes, we sought to investigate the potential relationship between these pathways in the context of HIV-associated neurodegeneration. Specifically, we hypothesized that HIV-1 Vpr might disrupt lysosomal positioning and degradative capacity through activation of CK1δ, leading to aberrant phosphorylation of SNAPIN. Our investigations into this Vpr-CK1δ-SNAPIN signaling axis using complementary molecular, imaging, and proteomic approaches provide insights into the mechanisms by which chronic HIV exposure contributes to neuronal damage and identify CK1δ as a potential therapeutic target for HANDs.

## Results

### HIV-1 Vpr drives lysosomal overload in neurons via impaired degradation of diverse cargoes

**Figure 1:**
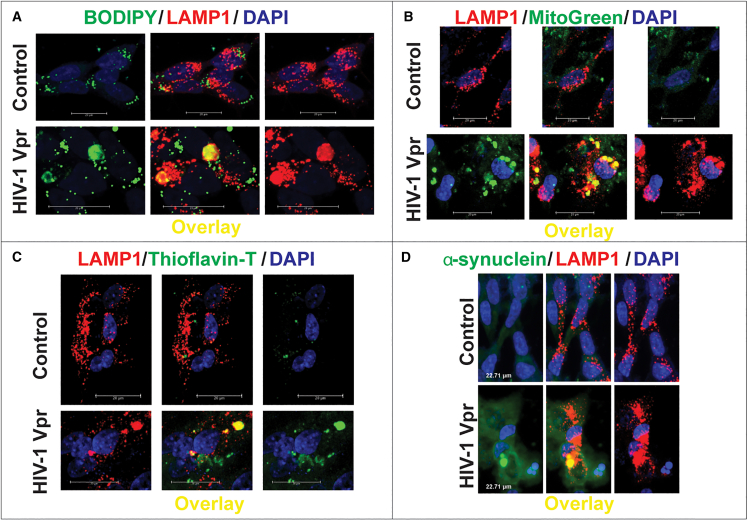
HIV-1 Vpr impairs lysosomal degradation, leading to cargo accumulation in neuronal lysosomes SH-SY5Y-derived neurons expressing LAMP1-mCherry were treated with recombinant HIV-1 Vpr (8 nM) and analyzed by confocal microscopy. (A) Lipid accumulation detected by BODIPY (green) colocalized with LAMP1 (red) after Vpr treatment. (B) MitoTracker-labeled mitochondrial remnants (green) persisted within lysosomes after Vpr exposure. (C) Thioflavin-T (green) revealed enhanced amyloid-like deposits in Vpr-treated lysosomes. (D) α-synuclein (green) showed increased lysosomal association following Vpr treatment. Scale bars: 20 μm. Representative qualitative images are shown; no statistical analysis was performed for this figure.

To determine whether HIV-1 Vpr disrupts lysosomal degradation in neurons, we treated SH-SY5Y cells differentiated into neurons with recombinant Vpr (8 nM) and assessed the clearance of diverse lysosomal cargoes by confocal microscopy. We observed a marked accumulation of lipid droplets, as revealed by BODIPY staining, which colocalized with the lysosomal marker LAMP1 (Figure 1A). Similarly, mitochondrial remnants retained MitoTracker signal even after CCCP-induced depolarization, indicating impaired mitophagy. These undegraded mitochondria accumulated in LAMP1^+^ compartments following Vpr exposure (Figure 1B).

In addition to metabolic substrates, we examined the fate of aggregated proteins. Thioflavin-T staining revealed increased amyloid-like deposits in Vpr-treated neurons, again colocalizing with lysosomes (Figure 1C). Moreover, SNCA, a protein implicated in neurodegeneration, showed enhanced lysosomal association following Vpr exposure (Figure 1D). Across all conditions, quantitative image analysis confirmed significantly elevated Manders’ colocalization coefficients between each cargo and LAMP1, consistent with their impaired degradation and entrapment within lysosomes.

After digitonin permeabilization, lysosome-enriched pellets and cytosolic supernatants were separated before substrate addition, ensuring that signals reflected intrinsic activity in each compartment (Figure S1). In controls, cathepsin activity—measured using the fluorogenic substrate Z-FR-AMC, which primarily reports cathepsin B and L activity—was largely confined to the pellet and declined gradually, while the supernatant showed only a modest rise from a low cytosolic baseline. Vpr accelerated the pellet decline and amplified the supernatant increase, indicating impaired intralysosomal proteolysis with redistribution of activity outside the lysosomal fraction. Inclusion of pepstatin A (aspartyl-cathepsin inhibitor) reduced reaction rates in both fractions but did not fully abolish them, consistent with partial inhibition of the mixed “pan-cathepsin” readout and confirming that the opposing kinetics reflect true compartmental activities rather than assay leakage.

Together, these data demonstrate that HIV-1 Vpr impairs lysosomal degradative capacity, leading to the accumulation of undigested lipids, mitochondria, and protein aggregates within neuronal lysosomes—a process that may contribute to neurodegeneration in HANDs.

### HIV-1 induces SNAPIN mislocalization across neuronal populations, with marked aggregation in cerebellar and Purkinje neurons

**Figure 2:**
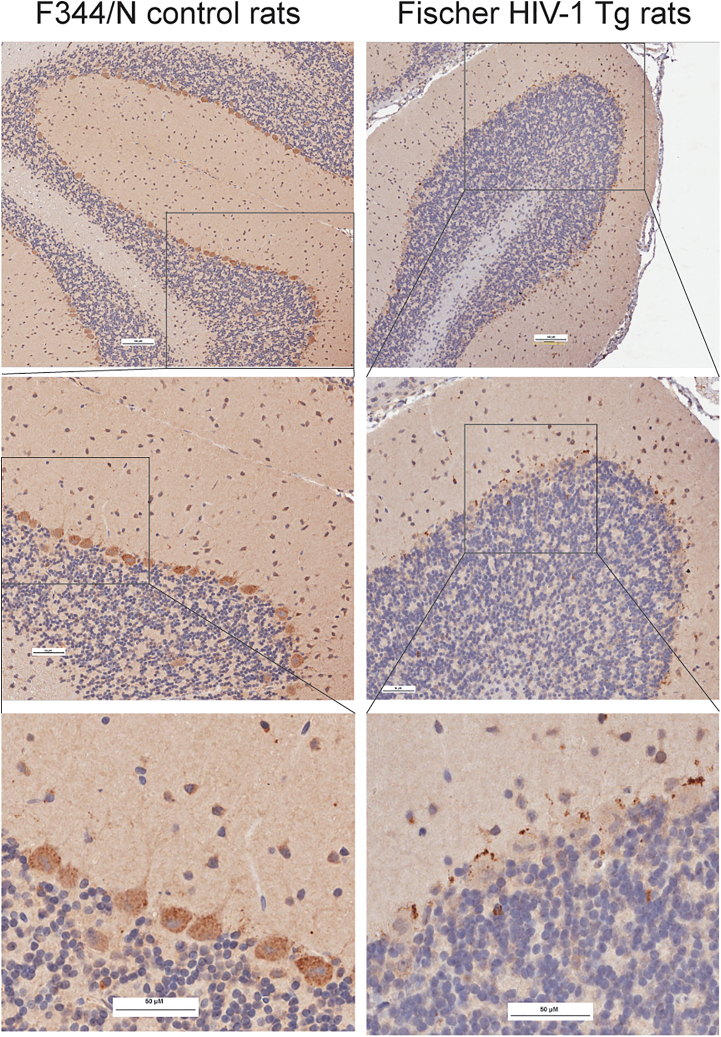
HIV-1 induces SNAPIN mislocalization and aggregation in neurons of HIV-1 transgenic rats Immunohistochemistry of cerebellar sections from control F344/N rats and HIV-1 Tg (Tg26) rats showed altered SNAPIN distribution. SNAPIN was diffuse in controls but accumulated perinuclearly in Purkinje neurons of HIV-1 Tg rats. Top: low magnification; middle: boxed regions; bottom: higher magnification of boxed areas showing SNAPIN clustering. No statistical tests were applied because this figure presents representative histology. Scale bars: 100 μm (top), 50 μm (middle/bottom).

To investigate whether HIV-1 Vpr affects the subcellular distribution of SNAPIN *in vivo*, we performed immunohistochemical analysis of brain sections from control and HIV-1 transgenic rats (Figure 2). In control F344/N rats, SNAPIN was diffusely expressed in the cytoplasm of Purkinje cells and molecular layer interneurons in the cerebellar cortex, without evidence of clustering. In contrast, HIV-1 transgenic brains displayed markedly increased SNAPIN signal, characterized by perinuclear clustering and punctate aggregation within Purkinje neurons and adjacent interneurons. Similar SNAPIN accumulation was also observed in extracerebellar regions, including the locus coeruleus, suggesting widespread dysregulation (data not shown). These findings indicate that HIV-1 Vpr drives abnormal SNAPIN accumulation and mislocalization in neurons, particularly in vulnerable cerebellar cell types.

### HIV-1 Vpr disrupts SNAPIN serine 50 phosphorylation, impairing lysosomal trafficking in neurons

**Figure 3:**
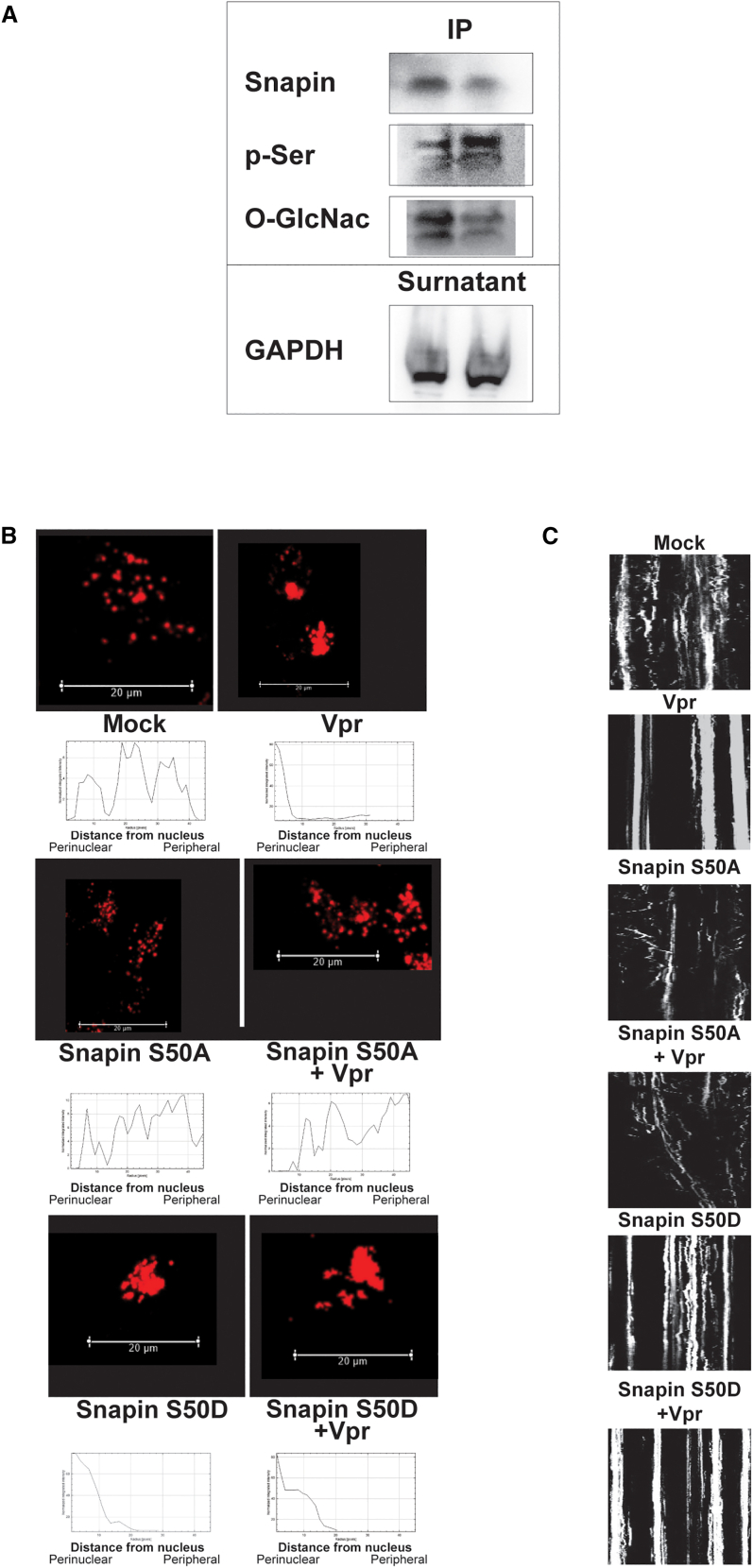
Vpr alters SNAPIN post-translational modifications and disrupts lysosomal trafficking via Ser50 (A) Immunoprecipitation of SNAPIN followed by western blotting showed increased serine phosphorylation and reduced O-GlcNAcylation after Vpr exposure (representative of *n* = 3 independent experiments). (B) Representative confocal images and radial intensity plots show Vpr-induced perinuclear lysosome clustering. S50D mimicked Vpr effects, whereas S50A prevented lysosomal clustering even in the presence of Vpr. (C) Kymograph analysis of LAMP1^+^ vesicle motility demonstrating reduced lysosomal movement with Vpr and S50D but preserved motility with S50A. Scale bars: 20 μm.

We previously reported that Vpr reduces SNAPIN expression, contributing to lysosomal mislocalization and impaired degradation in neurons.^25^ Here, we investigated whether HIV-1 Vpr also alters SNAPIN through posttranslational modifications. Immunoprecipitation followed by immunoblotting revealed that Vpr increases SNAPIN phosphorylation on serine residues while decreasing its O-GlcNAcylation—a modification associated with protein stability and trafficking function (Figure 3A).

To assess the functional relevance of SNAPIN phosphorylation, we systematically mutated all known phosphorylation sites identified in PhosphoSitePlus (www.phosphosite.org) and examined lysosome positioning in neurons. Of all the mutants tested, only alteration of serine 50 produced a distinct lysosomal phenotype, prompting focused analysis of SNAPIN serine 50 phospho-mutants to further characterize its role in lysosomal regulation. Vpr exposure induced prominent perinuclear clustering of lysosomes with reduced overlap with peripheral LAMP1^+^ vesicles, consistent with our previous findings,^25^ and this phenotype was recapitulated by the phosphomimetic S50D mutant (Figure 3B). In contrast, the non-phosphorylatable S50A mutant preserved normal lysosomal distribution even in the presence of Vpr.

Kymograph analysis in SH-SY5Y cells confirmed that lysosomal motility was impaired in both Vpr-exposed and S50D-expressing neurons, whereas S50A maintained directional trafficking (Figure 3C).

Comparable effects were observed in transdifferentiated neurons, where Vpr-induced lysosomal clustering and neurite disruption were both reversed by CK1δ inhibition with LH846 (Figure S2).

To determine whether CK1δ physically interacts with SNAPIN, we performed co-immunoprecipitation assays, which revealed increased CK1δ association with SNAPIN in Vpr-exposed neurons (Figure S3). This finding supports a direct kinase-substrate relationship enhanced by Vpr activity.

Together, these results identify serine 50 as a critical regulatory site through which Vpr impairs SNAPIN function, leading to defective lysosomal positioning and motility in neurons.

### HIV-1 Vpr induces CK1δ expression, leading to SNAPIN phosphorylation

Given that SNAPIN phosphorylation at serine 50 disrupts lysosomal positioning and motility in Vpr-exposed neurons, we next sought to identify the upstream kinase responsible. We performed a targeted pharmacological screen using inhibitors of kinases previously linked to SNAPIN regulation, including H-89 and Rp-8-Br-cAMPS (PKA), CID755673 (PKD), and LRRK2-IN-1 (LRRK2). Only inhibition of CK1δ with LH846 restored lysosomal motility and acidification, identifying CK1δ as the primary kinase driving SNAPIN phosphorylation in neurons. Among candidates, only the CK1δ inhibitor LH846 restored lysosomal function, prompting us to focus on CK1δ—a serine/threonine kinase known to regulate vesicle transport and neuronal signaling.

#### HIV-1 Vpr upregulates CK1δ expression

**Figure 4:**
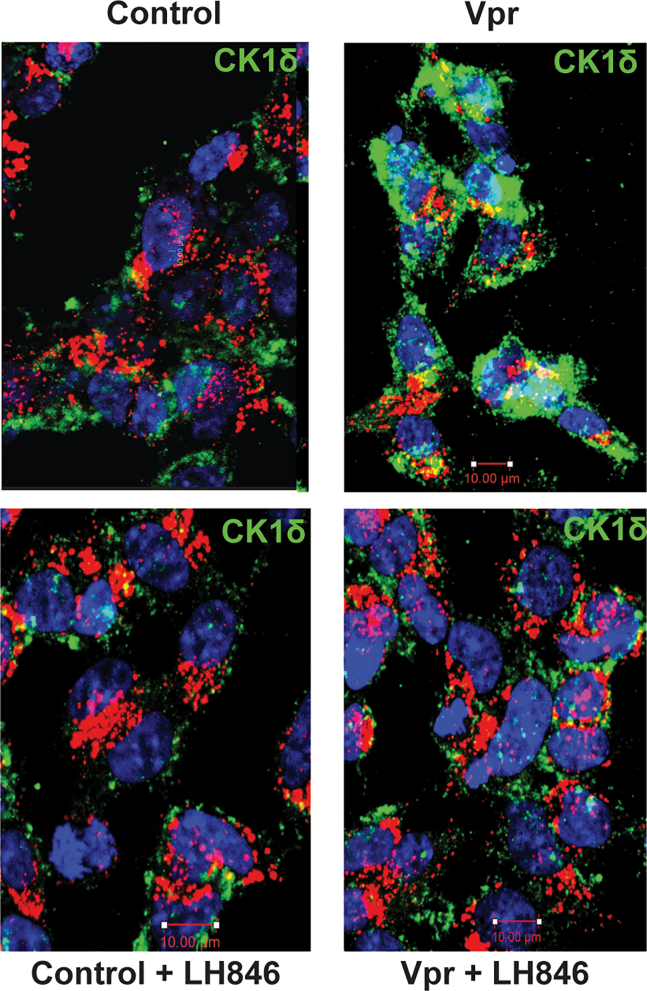
HIV-1 Vpr increases neuronal CK1δ expression Immunofluorescence of CK1δ (green) in SH-SY5Y-derived neurons treated with vehicle, Vpr, LH846, or Vpr + LH846. LAMP1 (red) labels lysosomes; nuclei are DAPI-stained (blue). Representative qualitative images are shown. Scale bars: 10 µm.

To explore whether CK1δ is regulated by Vpr, we assessed CK1δ expression by immunofluorescence (Figure 4). Vpr exposure resulted in increased CK1δ staining, which was partially reversed by LH846 treatment. Quantification confirmed that Vpr upregulates CK1δ expression in neurons, suggesting that Vpr may directly or indirectly activate this kinase as part of its pathogenic program.

#### CK1δ inhibition restores lysosomal positioning

**Figure 5:**
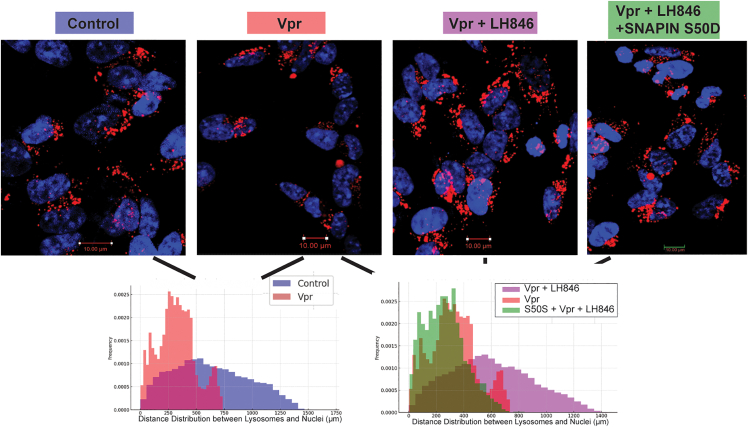
CK1δ inhibition restores lysosomal distribution disrupted by HIV-1 Vpr SH-SY5Y neurons expressing LAMP1-mCherry and SNAPIN wild-type (WT) or S50D were treated with Vpr ± LH846 (1.5 μM). Radial intensity profiles quantified lysosomal distance from nuclei. Scale bars: 10 μm.

To determine whether CK1δ contributes to lysosomal mispositioning, we assessed the spatial distribution of LAMP1^+^ vesicles in neurons treated with Vpr, with or without the CK1δ inhibitor LH846 (Figure 5). Control neurons displayed broadly distributed lysosomes, whereas Vpr exposure induced perinuclear clustering. LH846 restored peripheral lysosomal distribution, while co-expression of phosphomimetic SNAPIN (S50D) blocked this rescue. This finding underscores the functional importance of serine 50 in mediating CK1δ-dependent lysosomal clustering, linking CK1δ activity directly to SNAPIN phosphorylation at this site.

#### CK1δ inhibition restores SNAPIN localization

**Figure 6:**
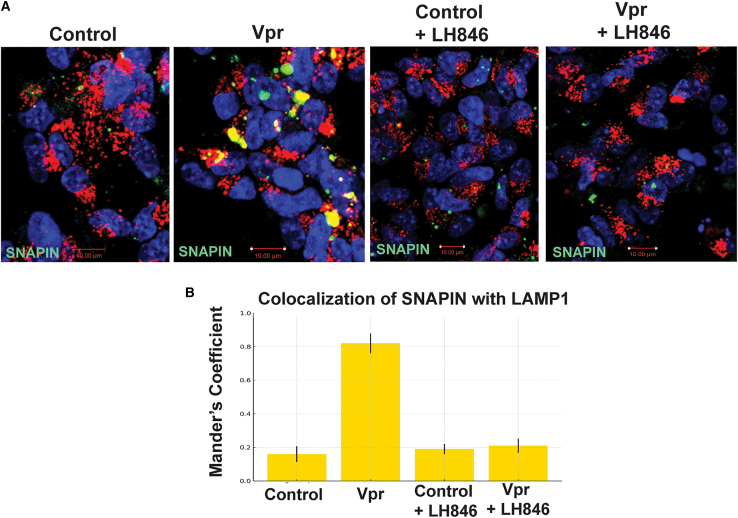
CK1δ inhibition restores SNAPIN distribution and lysosomal positioning (A) Representative immunofluorescence images of neurons expressing SNAPIN-GFP and LAMP1-mCherry treated with Vpr ± LH846. Images show SNAPIN relocalization following CK1δ inhibition. (B) Quantification of SNAPIN–LAMP1 colocalization using Manders’ coefficient. Colocalization increased with Vpr and was normalized by LH846 (mean ± SEM; *n* ≥ 100 cells from ≥3 independent differentiations). Statistics: one-way ANOVA with Tukey post hoc; *p* < 0.05. Scale bars: 10 µm.

We next tested whether CK1δ inhibition could reverse Vpr-induced SNAPIN mislocalization (Figure 6A). In Vpr-treated neurons, SNAPIN accumulated in clustered perinuclear aggregates and showed increased colocalization with LAMP1^+^ lysosomes—potentially explaining the overall reduction in detectable SNAPIN levels. Treatment with the CK1δ inhibitor LH846 restored a more diffuse cytoplasmic distribution of SNAPIN and significantly reduced its lysosomal association, as quantified by Manders’ coefficient (Figure 6B). These results indicate that CK1δ activity mediates Vpr-driven SNAPIN mislocalization and promotes its aberrant accumulation within lysosomes.

### CK1δ inhibition restores lysosomal acidification and mitophagy in Vpr-exposed neurons

**Figure 7:**
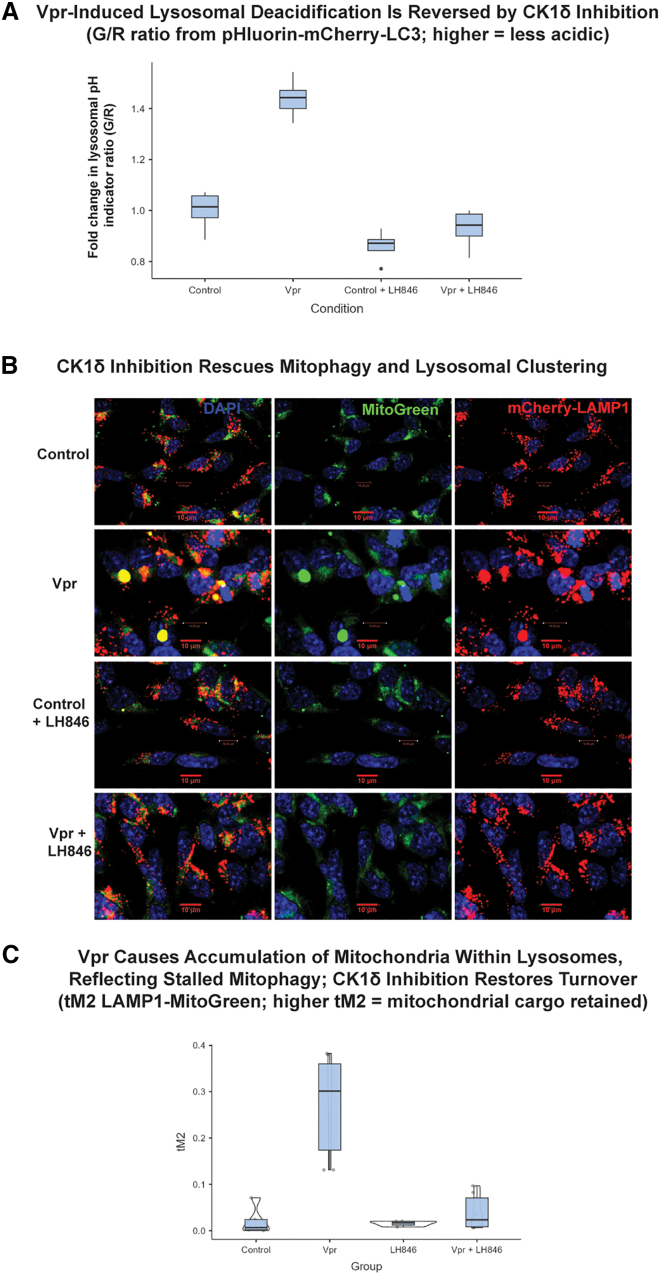
CK1δ inhibition restores lysosomal acidification and mitophagy in Vpr-exposed neurons (A) Lysosomal pH quantified using pHluorin-mCherry-LC3 (mean ± SEM; *n* ≥ 100 cells/condition from ≥3 independent differentiations). One-way ANOVA: F(3, 8.8) = 58.2, *p* < 0.001. (B) Representative confocal images of LAMP1 (red) and MitoGreen (green). Scale bars: 10 μm. (C) Colocalization (Manders tM2) between LAMP1 and MitoGreen (mean ± SEM; *n* ≥ 100 cells/condition from ≥3 differentiations). Welch ANOVA: F(3, 8.1) = 9.15, *p* = 0.006; Tukey post hoc *p* < 0.001 for Vpr vs. control and Vpr vs. Vpr + LH846.

To determine whether CK1δ inhibition rescues lysosomal function downstream of Vpr, we first assessed lysosomal pH using a dual-emission pH-sensitive LC3 reporter (pHluorin-mCherry-LC3) (Figure 7). Vpr exposure significantly increased the green/red (G/R) fluorescence ratio, consistent with lysosomal deacidification (Figure 7A). Treatment with the CK1δ inhibitor LH846 restored acidification to near-baseline levels, indicating that CK1δ activity contributes to Vpr-induced lysosomal alkalinization.

We next evaluated mitophagy and mitochondrial degradation by co-expressing a mitochondrial marker (MitoBright LT Green, simplified as MitoGreen) and a lysosomal marker (mCherry-LAMP1) (Figure 7B). In control neurons, damaged mitochondria were efficiently cleared, with minimal overlap between MitoGreen and LAMP1. In contrast, Vpr-exposed neurons displayed large accumulations of undegraded mitochondria that colocalized with LAMP1^+^ compartments, consistent with impaired mitophagy and lysosomal degradation. LH846 treatment markedly reduced MitoGreen retention and decreased LAMP1-mitochondria colocalization, as quantified by Manders’ coefficient (Figure 7C).

Together, these results demonstrate that CK1δ inhibition restores lysosomal acidification and degradative capacity in Vpr-exposed neurons, reversing mitochondrial and autophagic defects associated with SNAPIN misregulation.

## Discussion

Despite viral suppression with modern antiretroviral therapy, HANDs persist in a substantial subset of PLWH,^1^ underscoring the need to uncover virus-mediated, non-replicative mechanisms of neuronal dysfunction. Our study identifies a previously unrecognized signaling axis—Vpr-CK1δ-SNAPIN—that links HIV-1 exposure to lysosomal dysfunction, providing mechanistic insight into how chronic viral protein activity can disrupt organellar homeostasis and drive neurodegeneration.

We demonstrate that exposure to HIV-1 Vpr impairs lysosomal degradation by inducing the accumulation of undigested cargoes—including lipids, mitochondria, amyloid aggregates, and SNCA—within LAMP1^+^ compartments. These findings align with reports of endolysosomal abnormalities in HAND brain tissue^10^^,^^11^^,^^12^^,^^13^ and extend them by identifying SNAPIN misregulation as a central mediator of lysosomal trafficking failure. Specifically, we show that Vpr increases CK1δ expression in neurons, leading to SNAPIN hyperphosphorylation at serine 50, and mislocalization, possibly leading to functional uncoupling from motor complexes.^29^ This disruption in SNAPIN function impairs lysosomal positioning, motility, and degradative capacity—hallmarks of lysosomal stress previously reported in both HIV- and aging-associated neurodegenerative contexts.^10^

Importantly, our phosphomimetic SNAPIN mutant (S50D) recapitulates the Vpr-induced defects in lysosomal positioning and motility, while the non-phosphorylatable S50A mutant is protective. Pharmacological inhibition of CK1δ with LH846 reverses these effects—restoring SNAPIN localization, lysosomal acidification, and mitophagy—highlighting CK1δ as both an effector of Vpr toxicity and a promising therapeutic target in HANDs. LH846—a selective, ATP-competitive CK1δ inhibitor—was chosen for mechanistic studies because it affords cleaner CK1δ engagement than PF-670462 (δ/ε dual) and fewer off-target/cytotoxicity concerns than SR-3029 under neuronal conditions; accordingly, LH846 restored lysosomal pH, motility, and mitophagy in Vpr-exposed neurons, supporting CK1δ as a tractable target for HANDs. We propose a working model in which Vpr acts upstream of CK1δ, initiating a cascade that impairs neuronal clearance mechanisms through SNAPIN phosphorylation and lysosomal immobilization.

Our *in vivo* immunohistochemistry data extend these findings to the cerebellum, revealing that HIV-1 transgenic rats exhibit SNAPIN aggregation specifically in Purkinje cells and interneurons^42^—cell types increasingly recognized for their role in fine motor control,^43^ autonomic regulation,^44^ and sleep architecture.^45^ Purkinje neurons, in particular, are highly polarized and metabolically demanding, rendering them especially vulnerable to defects in organelle trafficking. Lysosomal dysfunction in these cells could therefore contribute to both the motor abnormalities (e.g., tremor and ataxia) and sleep disturbances frequently observed in HAND patients. Notably, neuroimaging studies in HIV+ individuals have reported significant reductions in frontal, brainstem, and cerebellar volumes in patients on integrase strand transfer inhibitor (INSTI)-based therapy compared to non-INSTI users,^46^ supporting a broader relevance of cerebellar vulnerability in HIV-associated neurodegeneration. Thus, our identification of a lysosome-targeting mechanism active in Purkinje neurons provides a potential cellular basis for diverse clinical symptoms in HAND. *In vivo*, the HIV transgenic (Tg26) and HIV Tg rat models exhibit motor incoordination, learning and memory impairments, and disrupted sleep architecture—behaviors consistent with the neuronal populations we identified as vulnerable to Vpr-induced lysosomal dysfunction.^47^^,^^48^ These phenotypes may reflect CK1δ-SNAPIN-mediated trafficking defects in Purkinje and brainstem neurons. Behavioral testing of CK1δ inhibition in these models is underway to determine whether restoring lysosomal function can improve cognitive and motor outcomes.

Mechanistically, our work builds on prior evidence linking CK1 family kinases to vesicle trafficking, proteostasis, and circadian biology.^49^ However, we are the first to implicate CK1δ as a Vpr-responsive kinase that phosphorylates SNAPIN and impairs lysosomal trafficking in neurons. This adds to a growing recognition that viral proteins can hijack host kinases to induce long-term cellular dysfunction, even in the absence of active viral replication.^50^^,^^51^ It also raises the possibility that CK1δ functions as a stress amplifier, integrating Vpr toxicity with broader disruptions in organellar homeostasis and clearance.

In this context, our data reveal that Vpr uncouples lysosomal positioning from acidification—a relationship normally maintained by microtubule-based transport and V-ATPase function. Although perinuclear lysosomes are typically more acidic in healthy cells, this property depends on continuous retrograde trafficking and proton-pump integrity. Vpr is thought to disrupt the CK1δ-SNAPIN-BORC-Arl8 machinery that coordinates motility with acidification, producing perinuclear but alkalized lysosomes. Since cathepsins are synthesized as inactive proenzymes that require progressive acidification and trafficking to reach their mature active forms, impaired transport and pH maintenance under Vpr exposure produce lysosomes with incomplete cathepsin activation and reduced degradative capacity.^52^ This mechanism explains the diminished cathepsin activity and lysosomal leakage we observe.

To extend these findings to a more physiological model, we used dopaminergic neurons directly transdifferentiated from human fibroblasts. This approach preserves donor-specific aging signatures lost in iPSC-derived neurons and enables modeling of HIV- and age-related neurodegeneration for further studies. Vpr again induced lysosomal clustering and deacidification, both rescued by LH846, supporting the relevance of the CK1δ-SNAPIN pathway across neuronal types and aging contexts.

In conclusion, we define a Vpr-CK1δ-SNAPIN signaling axis that underlies lysosomal dysfunction in neurons and contributes to HAND pathogenesis. These findings position CK1δ as a promising therapeutic target to restore neuronal degradative function in the context of chronic HIV exposure. Future studies will be needed to determine whether CK1δ inhibition can reverse cognitive deficits *in vivo* and to explore whether this axis plays a broader role in other neurodegenerative or lysosomal storage disorders where SNAPIN function is compromised.

### Limitations of the study

While this study establishes a mechanistic link between HIV-1 Vpr, CK1δ activation, and SNAPIN-mediated lysosomal dysfunction, several limitations warrant mention. First, most *in vitro* experiments used neuronal cell lines that enable precise mechanistic dissection but cannot capture circuit-level or behavioral outcomes; studies in animal models with cognitive and motor assessments will be essential to determine functional relevance. Second, our mechanistic conclusions rely on pharmacological CK1δ inhibition with LH846; complementary genetic loss-of-function approaches (siRNA/CRISPR knockdown of CK1δ) were not performed here and will be pursued to confirm causality and exclude residual off-target effects. Third, although LH846 effectively rescued Vpr-induced defects *in vitro*, its pharmacokinetics, brain penetrance, and long-term safety remain to be evaluated prior to translational application. Finally, although we demonstrate consistency in neurons directly transdifferentiated from human fibroblasts, broader validation in primary neuronal cultures and *in vivo* models will be important to strengthen the physiological relevance of the CK1δ-SNAPIN pathway in HANDs.

## Resource availability

### Lead contact

Further information and requests for resources and reagents should be directed to and will be fulfilled by the lead contact, Dr. Maryline Santerre (marylinesanterre@gmail.com).

### Materials availability

This study did not generate new unique reagents. Plasmids used in this work are available from Addgene under the identifiers listed in the key resources table. Fixed cerebellar tissues from HIV-1 transgenic and control rats were provided by Dr. Rosemarie M. Booze (University of South Carolina).

### Data and code availability

• No standardized datasets were generated for this study. All raw imaging files, processed data, and quantification outputs are available from the lead contact upon reasonable request.
• This study did not generate original code.
• Additional information required to reanalyze the data reported in this paper is available from the lead contact.

## Acknowledgments

This work was supported by an NIH-NIA grant AG054411 awarded to B.E.S. and an NIH-NIA grant R56AG082530 to M.S. We thank Kathy Q. Cai (Fox Chase Cancer Center) for performing the immunohistochemistry and Dr. Rosemarie M. Booze (University of South Carolina) for generously providing the rat brain tissue.

## Author contributions

M.S. designed the study, performed the experiments, analyzed the data, prepared figures, and wrote the manuscript. B.E.S. contributed to conceptualization, interpretation of results, and manuscript editing. All authors reviewed and approved the final manuscript.

## Declaration of interests

The authors declare no competing interests.

## Declaration of generative AI and AI-assisted technologies in the writing process

During the preparation of this work, the authors used ChatGPT to improve clarity, flow, and readability. AI was used solely for language editing; all content was reviewed and verified by the authors, who take full responsibility for the final manuscript.

## STAR★Methods

### Key resources table

**Table:** 

REAGENT or RESOURCE	SOURCE	IDENTIFIER
**Antibodies**		

Anti-SNAPIN antibody	Abcam	ab5669
Anti-CK1δ antibody	Cell Signaling Technology	#2655; RRID: AB_2283593
Anti-LAMP1 antibody	Abcam	ab24170
Anti-MAP2 antibody	Millipore	AB5622; RRID: AB_91939

**Biological samples**		

HIV-1 Tg rat cerebellar tissue	University of South Carolina	N/A
Control rat cerebellar tissue	University of South Carolina	N/A

**Chemicals, peptides, and recombinant proteins**		

LH846	Sigma-Aldrich	SML0985
CHIR99021	Sigma-Aldrich	SML1046
SB-431542	Tocris	1614
Forskolin	Sigma-Aldrich	F6886
LM22A-4	Tocris	5691
GDNF	PeproTech	450-10
bFGF	PeproTech	100-18B
MitoBright LT Green/Red	Dojindo	MT11-10
Recombinant HIV-1 Vpr protein	—	N/A

**Critical commercial assays**		

MycoAlert Mycoplasma Detection Kit	Lonza	LT07-418

**Experimental models: Cell lines**		

SH-SY5Y neuroblastoma cells	ATCC	CRL-2266
MRC-5 human fibroblasts (male)	ATCC	CCL-171

**Experimental models: Organisms/strains**		

HIV-1 Tg (Tg26) rats	University of South Carolina	N/A
F344/N control rats	University of South Carolina	N/A

**Recombinant DNA**		

pLKO-p53-shRNA-941	Addgene	#25637
tetO-ALN	Addgene	#43918
pPB-tetpA(hBCL2-miR-9/9∗-124)-iRPT	Addgene	#154881
pHluorin-mCherry-LC3	Addgene	#21074
pcDNA3.1-HA-SNAPIN WT/S50A/S50D	This study	N/A

**Software and algorithms**		

Fiji/ImageJ	NIH	v2.9.0
Jamovi	Jamovi Project	v2.5

### Experimental model and study participant details

#### Cell lines

SH-SY5Y neuroblastoma cells (ATCC CRL-2266) were maintained in DMEM/F12 supplemented with 10% fetal bovine serum (FBS) and 1× penicillin/streptomycin at 37°C and 5% CO_2_. Cells were authenticated by ATCC using STR profiling prior to distribution. Cells were tested monthly using MycoAlert (Lonza) and confirmed free of mycoplasma contamination. Cells were used between passages 5–25.

MRC-5 human fibroblasts (ATCC CCL-171) were maintained in DMEM/F12 with 10% FBS and used between passages 15–35. MRC-5 fibroblasts are derived from male fetal lung tissue. Mycoplasma testing was performed monthly and was consistently negative. Authentication was performed by ATCC.

#### Sex and gender in cell studies

Because the SH-SY5Y line is of cancer origin and MRC-5 is derived from a single fetal male donor, sex-based analyses were not applicable.

#### Primary and transdifferentiated human neurons

Neurons were generated from MRC-5 fibroblasts using a combination of lentiviral p53 shRNA knockdown and transposon-mediated expression of miR-9/9∗-124 and neuronal determinants. Fibroblasts were transfected with pPB-tetpA(hBCL2-miR-9/9∗-124)-iRPT and transduced with tetO-ALN and pLKO-p53-shRNA-941 in the presence of doxycycline (1 μg/mL). Cells were maintained in transdifferentiation medium (DMEM/F12, N2, B27, bFGF 20 ng/mL, GDNF 20 ng/mL, Forskolin 10 μM, LM22A-4 2 μM, CHIR99021 2 μM, SB-431542 10 μM, Vitamin C, and MDMH1) for 10–14 days.

#### Characterization

Cells were fixed and immunolabeled with TUJ1, neurofilament, MAP2, and TH antibodies. Imaging confirmed neuronal identity.

#### Human ethics

This work did not involve human subjects, identifiable human materials, or primary human tissue, and did not require IRB approval.

#### Animal models

Fixed cerebellar sections from HIV-1 Tg (Tg26) and control F344/N rats were obtained from Dr. Rosemarie M. Booze (University of South Carolina).

#### Animal ethics

Cerebellar tissue was obtained under protocols approved by the University of South Carolina Institutional Animal Care and Use Committee (IACUC), in accordance with NIH guidelines.

### Method details

#### Plasmid constructs and lentivirus production

Lentiviral particles encoding tetO-ALN and pLKO-p53-shRNA-941 were produced in HEK293T cells by co-transfection with psPAX2 and pMD2.G using Lipofectamine 3000. Viral supernatants were collected at 48 and 72 h, filtered, and used fresh or concentrated. Titers were determined by p24 ELISA. All lentiviral work was conducted under BSL-2 conditions approved by the Temple University IBC.

#### Vpr exposure and CK1δ inhibition

Recombinant HIV-1 Vpr (8 nM) was applied to neuronal cultures for 24 h. Rescue experiments used CK1δ inhibitor LH846 (1.5 μM, 24 h). Controls received DMSO (<0.1%).

#### SNAPIN mutant expression

SNAPIN WT, S50A, and S50D constructs were cloned into pcDNA3.1-HA and transfected with Lipofectamine 3000. Expression was validated via immunoblotting and immunofluorescence.

#### Lysosomal pH measurements

Lysosomal acidification was measured using the pHluorin-mCherry-LC3 reporter. The green/red (G/R) fluorescence ratio was quantified using Fiji.

#### Mitochondrial–lysosomal colocalization

Cells labeled with MitoBright LT Green and mCherry-LAMP1 were imaged by confocal microscopy. Manders’ coefficient tM2 was calculated using thresholded masks in Fiji.

#### Co-immunoprecipitation

Cells were lysed in RIPA buffer, incubated with anti-SNAPIN antibody and anti-HA beads, and analyzed by SDS-PAGE and immunoblotting.

#### Cathepsin activity assay

Lysosomal pellets and cytosolic supernatants were incubated with Z-FR-AMC substrate. Fluorescence kinetics were recorded over 60 min.

#### Immunohistochemistry

Rat cerebellar sections were antigen-retrieved, blocked, and incubated with anti-SNAPIN antibody. Alexa Fluor–conjugated secondary antibodies were used for detection.

### Quantification and statistical analysis

Image analyses were performed using Fiji/ImageJ v2.9.0 with Coloc2, Multi-Kymograph, and Radial Profile. n represents number of cells, independent differentiations, or tissue sections. Statistical analyses used Jamovi v2.5. ANOVA with Tukey post-hoc tests was applied. Data are reported as mean ± SEM, with significance set at p < 0.05.

### Additional resources

This study did not involve clinical trials and no additional resources are required.
