# Supplementary material for: CK1δ-dependent SNAPIN dysregulation drives lysosomal failure in HIV-1 Vpr-exposed neurons: A targetable mechanism in HAND
Source: iScience. 2025 Dec 26;29(2):114544. doi: 10.1016/j.isci.2025.114544 (PMC12818168; doi:10.1016/j.isci.2025.114544)
Supplement: Document S1. Figures S1–S3 [file mmc1.pdf]

iScience, Volume *29*

Supplemental information

CK1δ-dependent SNAPIN dysregulation drives lysosomal failure in HIV-1 Vpr-exposed neurons: A targetable mechanism in HAND

Bassel E. Sawaya and Maryline Santerre

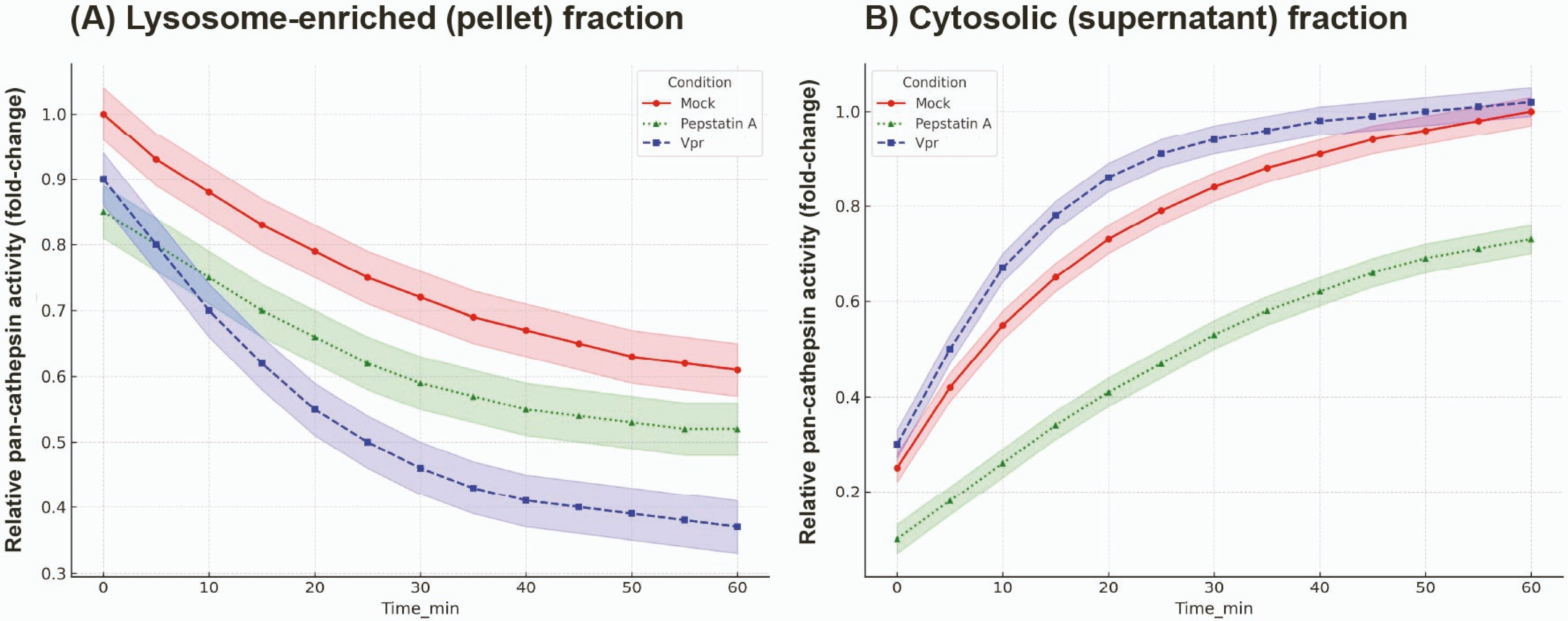

Figure S1. HIV-1 Vpr reduces lysosomal cathepsin activity and promotes cytosolic leakage.

(A)

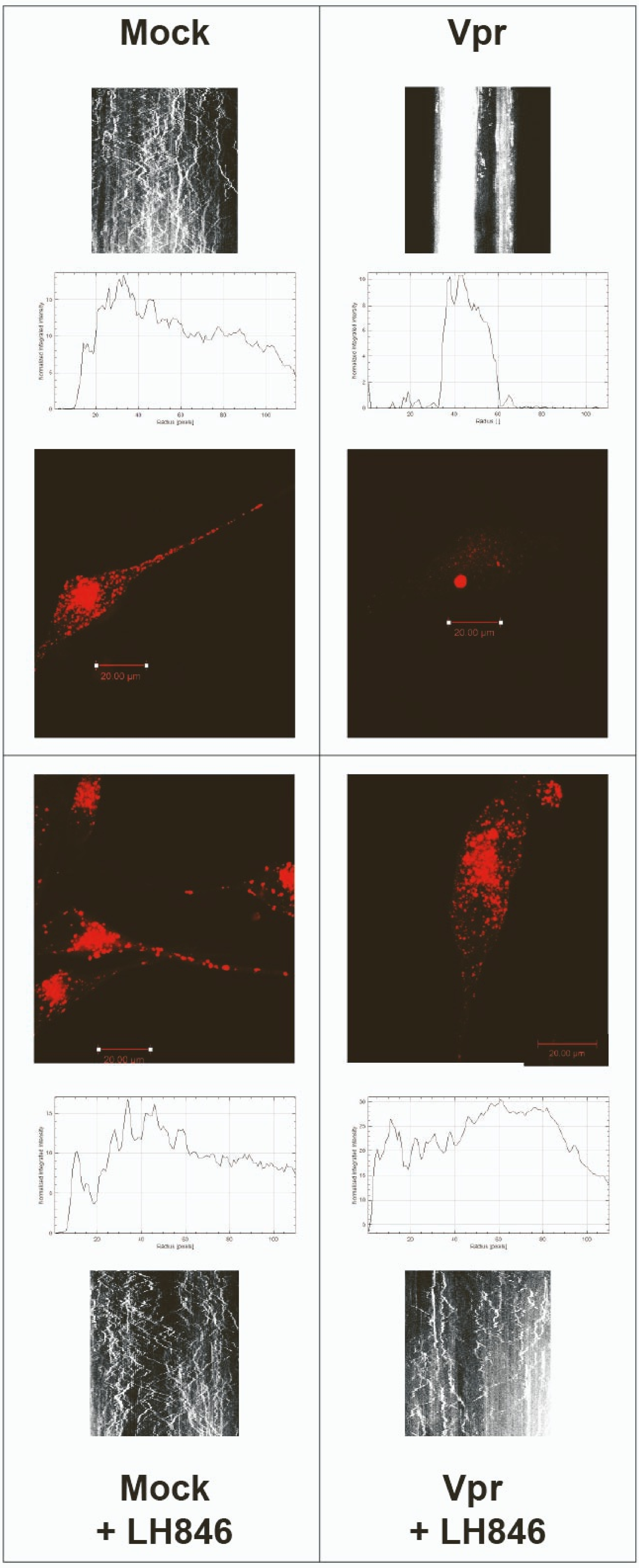

(B)

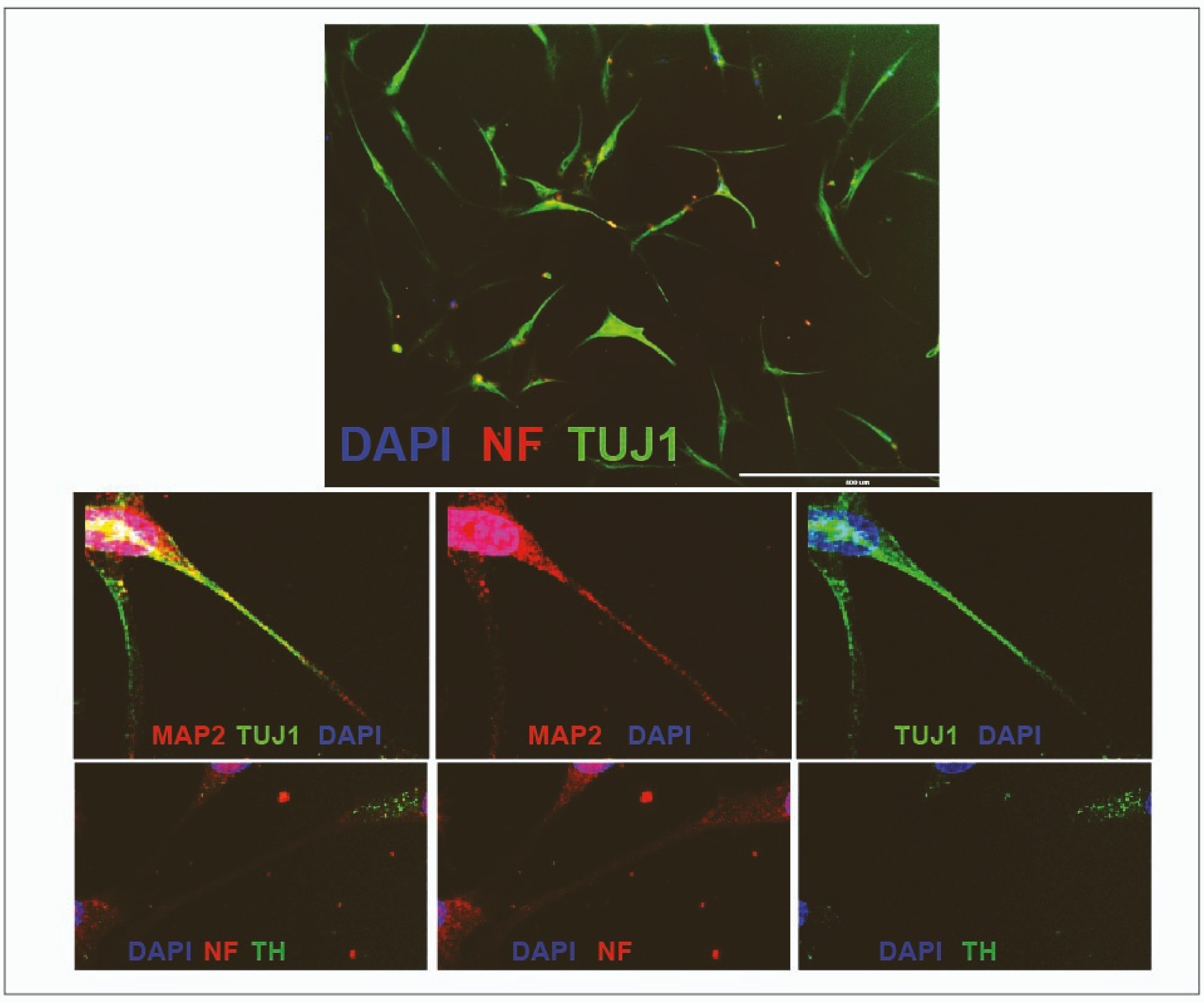

Figure S2. LH846 restores lysosomal distribution and neurite integrity in Vpr-exposed transdifferentiated neurons.

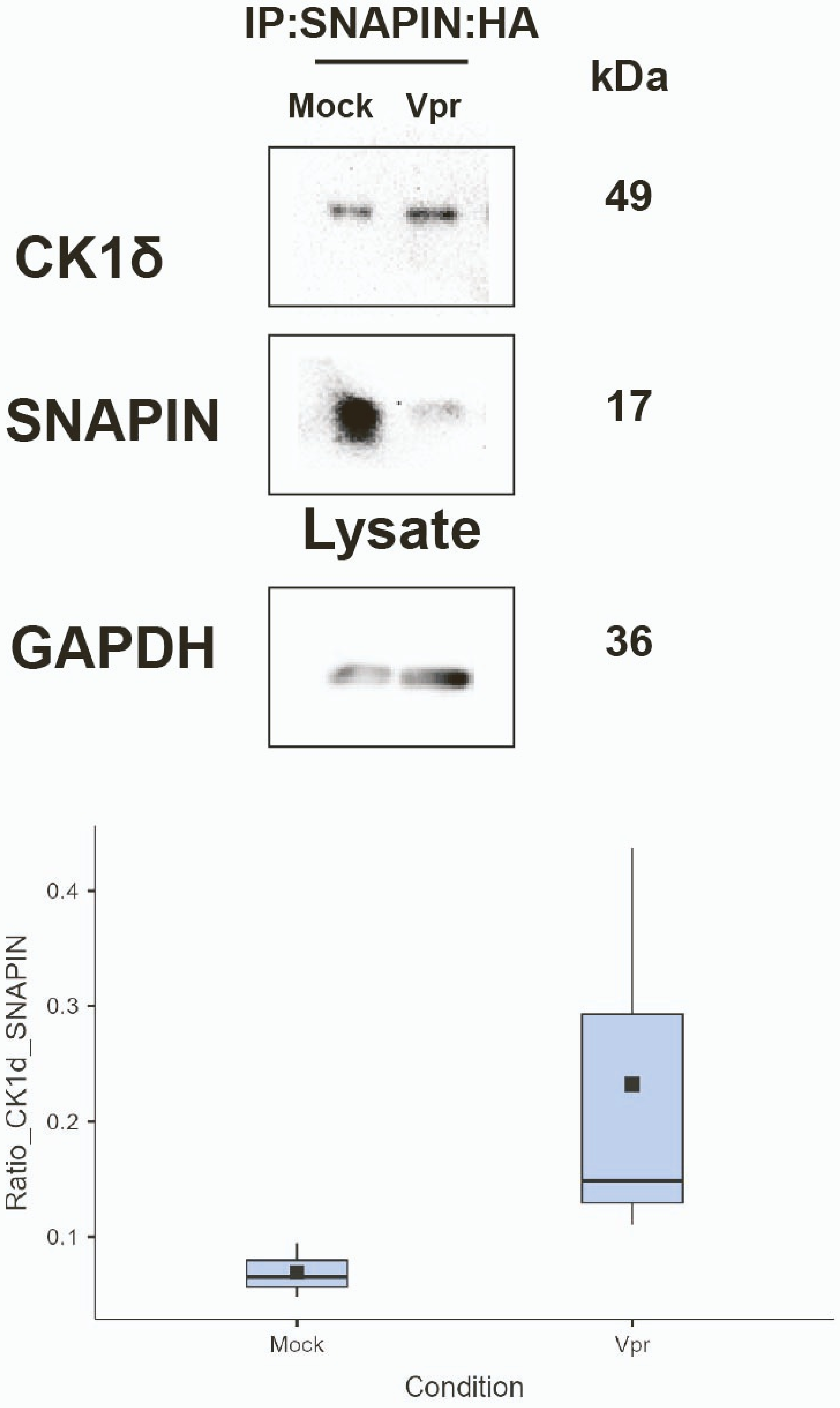

Figure S3 HIV-1 Vpr enhances the interaction between CK1δ and SNAPIN.

Supplementary Legends

Figure S1. HIV-1 Vpr decreases lysosomal cathepsin activity and promotes cytosolic leakage.

(A) Time-course of lysosomal cathepsin activity in pellet fractions.
(B) Cytosolic fractions showing increased cathepsin release after Vpr.
Data are **mean ± SEM; n = 3 independent experiments**.

Figure S2. LH846 restores lysosomal distribution and neurite integrity in Vpr-exposed transdifferentiated neurons.

(A) Lysosomes labeled with Lyso-ID and analyzed using radial profiles and kymographs.
(B) Characterization of transdifferentiated neurons by TUJ1, NF/MAP2, TH staining.
Representative images only (no statistics).
Scale bars: 20 µm (A), 400 µm (B).

Figure S3. HIV-1 Vpr increases CK1δ association with SNAPIN.

Co-IP of HA-SNAPIN with CK1δ in neurons ± Vpr.
Quantification of CK1δ/SNAPIN ratios (**mean ± SEM; n = 3 independent experiments**).
GAPDH loading control included.
